# Genome Sequence of the Sulfate-Reducing Bacterium Pseudodesulfovibrio portus JCM 14722^T^

**DOI:** 10.1128/mra.00947-22

**Published:** 2022-11-21

**Authors:** Takafumi Kataoka, Ryuji Kondo

**Affiliations:** a Department of Marine Science and Technology, Fukui Prefectural University, Obama, Fukui, Japan; Wellesley College

## Abstract

Pseudodesulfovibrio portus JCM 14722^T^ is a strictly anaerobic, mesophilic sulfate-reducing bacterium isolated from estuarine sediments in Japan. Its draft genome sequence comprises 1 circular chromosome (3,403,863 bp), harboring 3,182 predicted protein- and 60 tRNA-encoding genes, as well as 2 rRNA operons.

## ANNOUNCEMENT

Dissimilatory sulfate-reducing bacteria are obligate anaerobic prokaryotes which play a significant role in the biogeochemical cycling of sulfur and carbon in anoxic environments (1, 2). The sulfate-reducing bacterial genus *Pseudodesulfovibrio* includes 11 Gram stain-negative, mesophilic species that are characterized by dissimilatory sulfate reduction with incomplete oxidation of low-molecular-weight fatty acids (3–5). The genomes of all the type strains of the *Pseudodesulfovibrio* species have been sequenced, except for that of Pseudodesulfovibrio portus JCM 14722^T^ (=MSL79^T^), which was isolated from estuarine sediments in the Sea of Japan (6). Therefore, no data have been available on the *P. portus* genome or the levels of DNA relatedness between *P. portus* and other *Pseudodesulfovibrio* species.

An actively growing culture of *P. portus* JCM 14722^T^ was supplied by the Japan Collection of Microorganisms (JCM), RIKEN BioResource Research Center. It was inoculated into a 1-L anaerobic culture bottle (Sanshin) containing 500 mL of DSMZ 195c medium (https://www.dsmz.de/microorganisms/medium/pdf/DSMZ_Medium195c.pdf) and incubated at 30°C in the dark. Next, the cell pellet was harvested by centrifugation at 3,310 × *g* and 4°C for 30 min. The standard phenol-chloroform extraction method was used to extract genomic DNA (gDNA) from the cell pellet, as described previously (7). After the final purification of the extracted DNA using a short-read eliminator kit (Circulomics), the gDNA was sheared to 10- to 20-kb fragments using a g-TUBE device (Covaris). A long-read DNA library was constructed for strain JCM 14722^T^ using the SMRTbell Express template prep kit v2.0 (PacBio), following the manufacturer’s instructions, and sequenced using the PacBio Sequel IIe system. High-fidelity reads (total, 20,526) were obtained from the generated subreads using circular consensus sequencing via SMRT Link v10.1.00.119528. Then, 90% of the best-quality reads containing >1,000 bp were screened using Filtlong v0.2.0 (https://github.com/rrwick/Filtlong), and the remaining 18,361 reads (3,068 to 40,961 bp) were used for *de novo* assembly, which was performed using Flye v2.8.3 (8), with a minimum overlapping setting of 5,000 bp under uneven coverage mode. Default parameters were used for all software unless otherwise specified. This resulted in a single 3,403,863-bp circular genome with 57× coverage and an overall G+C content of 61.2%, which is slightly lower than that measured using HPLC (6).

The genome was annotated using DFAST (9), available at the DNA Data Bank of Japan (DDBJ). The draft genome sequence comprised 2 rRNA operons, 60 tRNA genes, and a total of 3,182 coding DNA sequences, including 1,267 genes encoding hypothetical proteins. Within the single genome, two 16S rRNA gene sequences were found to be identical.

As no data are available on the DNA-DNA reassociation between *P. portus* JCM 14722^T^ and other *Pseudodesulfovibrio* species, digital DNA-DNA hybridization (dDDH) values were calculated using formula 2 of the GGDC tool (http://ggdc.dsmz.de/distcalc2.php) (10). The closest relative of *P. portus* JCM 14722^T^ was identified as Pseudodesulfovibrio mercurii ND132^T^, with a dDDH value of 22.7%, followed by Pseudodesulfovibrio hydrargyri BerOc1^T^, with a dDDH value of 22.4%.

### Data availability.

The genome sequence of *P. portus* JCM 14722^T^ has been deposited at the DDBJ under the accession number AP026708. The raw reads have been deposited in the DDBJ Sequence Read Archive under accession number DRA014801. The BioSample and BioProject accession numbers are SAMD00520464 and PRJDB14192, respectively.
